# Interventions targeting challenges experienced by individuals with Pitt Hopkins syndrome: a scoping review

**DOI:** 10.1186/s13023-026-04561-6

**Published:** 2026-08-27

**Authors:** Monika Dolik-Michno, Magnus Starbrink, Helena Wandin, Annika Jernberg Grönlund, Martha Gustavsson, Linn Johnels

**Affiliations:** 1 Swedish National Center for Rett Syndrome and Related Disorders, Frösön, Sweden; 2 Oslo Metropolitan University, Oslo, Norway; 3 Department of Public Health and Caring Sciences, University of Uppsala, Uppsala, Sweden; 4 Department of Community Medicine and Rehabilitation, Umeå University, Umeå, Sweden; 5 Department of Women’s and Children’s Health, University of Uppsala, Uppsala, Sweden; 6 Department of Education and Special Education, University of Gothenburg, Gothenburg, Sweden; 7 Nationellt Center för Rett Syndrom & Närliggande Diagnoser, Box 601, Frösön, 832 23 Sweden

**Keywords:** Pitt–Hopkins syndrome, Rare disease, Intervention study, Epilepsy management, Breathing abnormalities

## Abstract

**Background:**

Pitt–Hopkins syndrome (PTHS) is a rare neurodevelopmental disorder with pronounced impacts on physical health and everyday functioning, including severe intellectual disability, impaired communication, epilepsy, and breathing dysregulation. Despite significant clinical challenges, there is limited evidence to guide interventions for individuals with PTHS. This scoping review aimed to examine the available literature on interventions targeting challenges experienced by individuals with PTHS and to identify gaps in the current knowledge base.

**Results:**

Sixteen peer-reviewed publications, published between 2012 and 2024, met the inclusion criteria. The majority of studies focused on medical management, predominantly addressing epilepsy and breathing abnormalities, and were pharmacological in nature. Most studies employed case report or small case series designs, and systematic outcome measurement approaches were rare. Epilepsy interventions largely reflected clinical practices in broader epilepsy populations, with common use of conventional antiseizure medications and evidence of potential benefit from newer agents in treatment-resistant cases. Non-pharmacological treatments, including vagus nerve stimulation, corpus callosotomy, ketogenic diet, and a walking-based mobility intervention, were described in only a few studies, with heterogeneous outcomes. Interventions for respiratory dysfunction, gastrointestinal symptoms, pain management, immune dysfunction, psychiatric or behavioral difficulties were limited and typically reported in individual cases. Research addressing psychosocial, behavioral, and participation-focused interventions was notably scarce, despite the broad functional impairments associated with PTHS.

**Conclusions:**

This scoping review shows that intervention research in PTHS remains at an early stage, with an evidence base dominated by descriptive case studies and few systematic evaluations. This limits the ability to formulate robust, evidence-based treatment recommendations. There is a need for more rigorous research designs in future research, that enable causal inference and systematic outcome measurement. Moreover, future studies should broaden the scope of intervention research to include communication, behavior, participation, physical activity and quality of life, alongside medical management, to better support the complex needs of individuals with PTHS. Increased focus on family impact and long-term outcomes may further enhance understanding and care strategies for this population.

## Background

Pitt-Hopkins’ syndrome (PTHS) is a rare autosomal dominant syndrome caused by a mutation or deletion of gene TCF4 localised on the long arm of chromosome 18. Diagnosis is based on clinical symptoms, with particular emphasis on facial appearance, intellectual disability with impaired communication, and abnormal breathing regulation. Although the prevalence is unknown, it is estimated to 1 in 300 000 [1]. The mutation occurs in males and females and the risk of the mutation being hereditary is low. Two, extremely rare, Pitt Hopkins’-like syndromes exist. These are caused by mutations in gene CNTNAP2 on chromosome 7 and gene NRXN1 on chromosome 2 respectively with a higher risk of heredity.

Abnormal breathing during wakefulness, characterized by recurrent episodes of hyperventilation, has been reported in approximately 48% of individuals with PTHS. These characteristic episodes are often followed by central apnea and may be followed by lip cyanosis and occasionally even fainting [2]. Abnormal breathing episodes may begin at varying ages, often occurring alongside or near the onset of epilepsy [3], and are seen in nearly 90% of older individuals. There is no correlation between the abnormal breathing patterns and the type of genetic mutation, nor with electroencephalographic (EEG) changes during the episodes that would suggest an epileptic origin. Abnormal breathing during sleep is rarely reported and seldom investigated using EEG or polysomnography (PSG) [4]. As of today, there is no recommended treatment.

Epilepsy is another common neurological manifestation in individuals with PTHS, with reported prevalence rates ranging from 37% to 50% [5]. Seizure onset occurs at variable ages, and multiple seizure types have been described [3, 6]. Early-onset epilepsy, particularly during infancy or early childhood, is frequently associated with Developmental and Epileptic Encephalopathy, characterized by profound intellectual disability, marked developmental delay, and in some cases, developmental regression. To date, no correlations have been found between seizure type, mutation characteristics, EEG findings, neuroimaging abnormalities, and epilepsy severity. Notably, a later onset of epilepsy may be associated with a more favorable developmental outcome. More than half of the individuals with PTHS and epilepsy demonstrate a favorable response to anti-seizure medications (ASM) [5]; however, no specific medication has been universally recommended.

Other common symptoms that may indicate PTHS include myopia, motor difficulties with limited walking ability and ataxia, gastrointestinal issues such as constipation, and minor structural brain abnormalities [1].

Structural brain abnormalities are among the most frequently observed anomalies in PTHS, present in approximately 60–70% of the individuals. The most common findings include hypoplasia or aplasia of the corpus callosum, ventriculomegaly, and posterior fossa malformations, such as a thin hindbrain [7]. Malformations of the genitourinary system have been documented in approximately 30% of the cases [1, 2]. Gastrointestinal anomalies, such as intestinal agenesis and malrotation, are rare but may lead to serious complications. Skeletal anomalies in individuals with PTHS predominantly affect the feet and commonly present as pes planus, frequently accompanied by valgus deviation (57%). Additional abnormalities include overriding toes (43%) and clubfoot (20%). Scoliosis has been reported in approximately 18% of individuals and typically develops during puberty, although onset may occur earlier in some cases [1, 2]. Individuals with PTHS typically exhibit moderate or severe intellectual disability and expressive language impairment [1]. Only approximately 20% have achieved partial control over bladder and bowel function, and lifelong dependence on caregivers is universal. However, rare cases have been reported in which individuals with pathogenic variants in the TCF4 gene present with milder phenotypes and even typical cognitive development [1].

Motor milestone attainment is frequently delayed, with approximately 75% of affected individuals achieving independent ambulation between 6 and 10 years of age. Gait is typically broad-based, unstable, and ataxic. Tremor has been reported in a minority of cases. Muscle tone is variable; hypotonia is observed in approximately 76%, predominantly involving truncal musculature, while hypertonia is reported in about 7% of the cases. Additionally, peripheral hypertonia has been documented in approximately 34% [6].

Moreover, behavioral phenotyping indicates a broad spectrum of neuropsychiatric features. While many individuals are described as cheerful and sociable (51%), behavioral challenges are common. These manifestations include aggressive behavior (48–54%), self-injurious behavior (70.8%), impairments in social interaction, anxiety, agitation, repetitive behaviors, and features consistent with the autism spectrum disorder (ASD) (41.7%) [6, 8].

Due to the complexity and severity of their symptoms, people with PTHS require support throughout their lives. The serious medical conditions, in many cases, require careful long-term management. Moreover, parents of people with PTHS have reported that aspects of daily living, including independence and the capacity to participate in everyday activities may be important for their children’s quality of life [9]. Although some studies are currently underway, there remains a paucity of intervention studies to guide clinical care. Zollino et al. [1] have published consensus statements on diagnosis and management drawing on evidence from broader populations as well as clinical experience. At the time of writing, clinical practice guidelines are being developed using a consensus methodology by the ERN ITHAKA Clinical Guidelines Workgroup [10]. To the best of our knowledge, there is currently no published review of studies aimed at improving health and daily life functioning among individuals with PTHS.

To address the need for evidence-based recommendations and to inform future research, we conducted this scoping review to examine the existing literature on all kinds of interventions targeting challenges experienced by individuals with PTHS and to identify gaps in the current knowledge base. Specifically, this review aimed to examine:

- What are the characteristics of the participants in the studies?
- Which study designs and methods have been used?
- What types of interventions have been performed targeting challenges of individuals with PTHS?
- To what extent are the interventions reported as beneficial or effective?
- What is the scientific quality of the included studies?

## Method

To examine the literature on a broad definition of interventions targeting challenges experienced by individuals with PTHS, this study was designed as a scoping review. The process of identifying and selecting relevant articles was executed in accordance with the PRISMA guidelines [11] and the PRISMA extension for scoping reviews [12]. On June 17, 2024, this review was registered with the Open Science Framework (https://doi.org/10.17605/OSF.IO/CPW2K).

### Search strategy

The search strategy was launched in October and November 2023 by two of the authors. The search was conducted by the two authors independently in databases Medline (PubMed), CINAHL and ERIC. Due to accessibility issues, search in PsycInfo was ran solely by one of the authors. In September 2025, an additional search was launched to identify more recently published research. Search strategy details are displayed in Table 1. Search results were exported to Endnote and after removal of duplicates by two of the authors, the references were imported to the free web-based RAYYAN platform (https://rayyan.qcri.org) for further collaborative screening and selection procedures.

**Table 1:** Search strategies by database

Medline (Oria), PsycInfo	Medline (PubMed)	CINAHL/ERIC
“pitt-hopkins syndrome”.mp. OR “pitt-hopkins”.mp. OR “pitt hopkins syndrome”.mp. OR "pitt hopkins”.mp. AND activ*.mp. OR “Activities of Daily Living”/ or “daily living”.mp. OR educati*.mp. OR therap*.mp. OR program*.mp. OR support.mp. OR exp Counseling/ or counseling.mp. OR management.mp. or exp Management/ OR training.mp. or exp Training/ exp Treatment/ or treatment.mp. OR intervention.mp. or exp Intervention/ coaching.mp. or exp Coaching/disease management.mp. or exp Disease Management/ OR “daily activities”.mp. or exp Daily Activities/	((((((“pitt-hopkins syndrome”)) OR (“pitt-hopkins”)) OR (“pitt hopkins syndrome”)) OR (“pitt hopkins”))) AND (((((((((((((((management) OR (activit*)) OR (“daily living”)) OR (educati*)) OR (training)) OR (treatment)) OR (intervention)) OR (therap*)) OR (program*)) OR (support)) OR (coaching)) OR (counseling)) OR (disease management)) OR (“activities of daily living”)))	pitt-hopkins syndrome OR pitt-hopkins OR pitt hopkins syndrome OR pitt Hopkins

Note: In Medline (Oria), PsycInfo and Medline (Pubmed) filters human and English language were applied

### Selection of relevant research articles

To identify all interventions described in the literature, the inclusion criteria were intentionally broad, for instance with respect to study design. However, to ensure an acceptable level of methodological quality, only peer-reviewed articles were included. Studies involving individuals with Pitt–Hopkins–like syndrome were excluded to ensure a more homogeneous study population. Detailed inclusion and exclusion criteria are presented in Table 2. Following calibration of the interpretation of the inclusion and exclusion criteria, two authors independently conducted an initial screening to exclude clearly irrelevant records (e.g., studies involving non-human subjects, biological material, or computer-generated models not captured by the search filters). Subsequently, the same two authors independently screened titles and abstracts to identify articles eligible for full-text review according to the predefined criteria. Articles deemed potentially eligible were distributed among all authors for an initial full-text assessment, preliminary data extraction, and recommendations regarding inclusion or exclusion. Any uncertainties were resolved through consensus discussions involving all authors. To identify recently published studies, the search strategy was replicated independently by two authors, and the results were screened in accordance with the same procedures. Finally, the reference lists of all included studies were manually reviewed by one author to identify additional relevant publications.

**Table 2:** Criteria for inclusion and exclusion

	Subjects	Interventions	Additional
Inclusion Criteria	Studies including at least one (1) individual with PTHS (both clinically and genetically confirmed diagnosis). Studies on living humans.	Studies that evaluate intervention or report any extractable outcome data for individual(s) with PTH.	Peer reviewed studies. All designs. All years. Written in English.
Exclusion Criteria	Studies with no individual with PTHS. Studies with individuals with PTHS-like syndrome. Studies on animals. Studies on body tissue, blood samples etc. Studies on computer models.	Studies targeting only method or strategy development.	Written in other language than English. Review article.

### Data extraction and quality appraisal

Two of the authors examined the eligible articles and sorted data into a data extraction chart. Three of the authors evaluated the included articles using the JBI checklists for case reports and case series studies [13, 14], with each article being evaluated twice.

### Inter-rater reliability

Inter-rater reliability for screening and eligibility of articles was calculated and computed in Excel, using the following formula: number of agreements divided by total number. Overall, the agreement across raters was substantial with an IRR of 97% for ruling out obviously irrelevant articles, an IRR of 93% for title and abstract screening and an IRR of 96% for full text screening.

For quality appraisal, intraclass correlations (ICCs) with absolute agreement and values for a single measure were calculated for the total JBI quality appraisal score. The obtained ICCs ranged from 0.82 to 1.00, corresponding to excellent IRR according to criteria in Cicchetti [15].

## Results

**Fig. 1:**
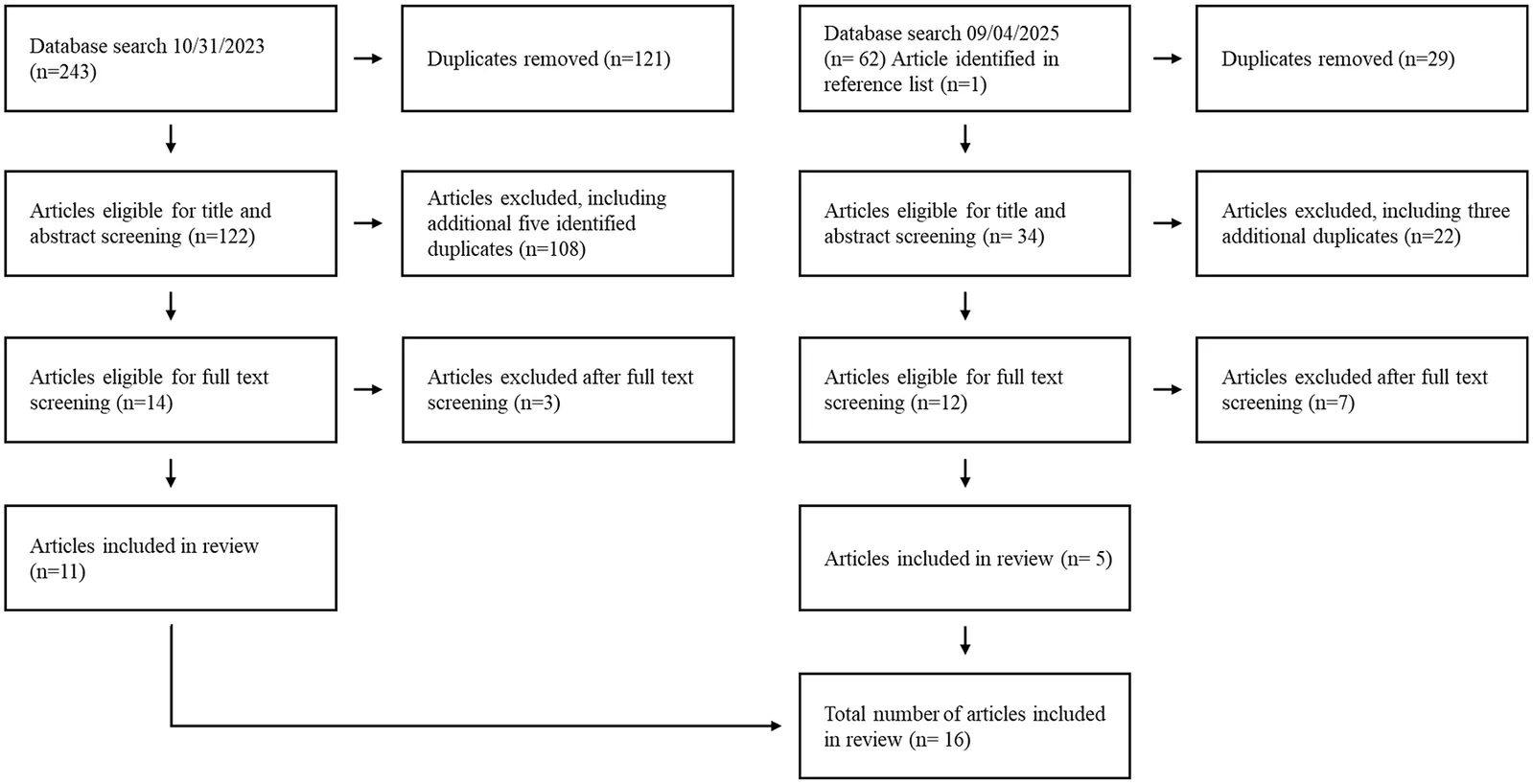
PRISMA flow diagram of the initial and complementing search and screening processes in PubMed, Medline, PsycInfo, CINAHL, and ERIC

The initial search yielded 243 articles, with an uneven distribution across the databases (i.e., CINAHL *N* = 20, ERIC *N* = 1, Medline *N* = 100, Medline PubMed *N* = 107 and PsycInfo *N* = 15). An additional search identified another 62 articles (CINAHL *N* = 12, ERIC *N* = 0, Medline *N* = 23, Medline PubMed 25 = X and PsycInfo = 2). In addition, one article was identified in a review of reference lists. After duplicate records were removed using EndNote, the remaining articles were imported into Rayyan (https://rayyan.qcri.org) for further screening. The complete results of the screening process are presented in Fig. 1.

In total, 16 articles met the inclusion criteria and were included in the review. Table 3 presents an overview of participant characteristics, study designs, comorbid conditions, intervention effectiveness, quality appraisal, as well as the country of origin, publication year, and authorship of the included studies.

**Table 3:** Summary of the included studies organized by intervention target

Author, Year, Study design, Country,	Participants	Symptoms and comorbidities^1^	Intervention	Reported Effects or Benefits	Quality Appraisal^1^ JBI rating (%)
**Epilepsia**					
Calle-Lopez et al., 2019. Case report. Brazil.	1 female. Age: 13 years. Genetically confirmed PTHS diagnosis.	Refractory epilepsy with 3–4 seizures yearly. Stereotypies.	ASM. Introduction of PMP in addition to VPA after several non-satisfactory medical try-outs. Follow-up at 9 months after the start of the intervention.	Improvement of seizure control. Recurrence of seizures after 9 months. Curly hair as side-effect of PMP. Outcome measure not reported.	88/100 Demographic characteristics and assessment methods: not sufficiently described.
Kirikae et al., 2022. Case report. Japan.	1 male. Age: 2 years Genetically confirmed PTHS diagnosis.	Migrating partial seizures within the first month of life, of infancy, and at the age of ten months. Infantile spasm with hypsarrhythmia (West syndrome). Increase of symptom severity following seizures debute. Stereotypies.	ASM. Introduction of TPM after several non-satisfactory medical try-outs. Follow-up: 17 months after start of intervention.	Complete seizure control. Improvement of background activity and epileptic discharges. Improvement of psychomotor function. Outcome measure reported; EEG	88 No adverse effects reported.
Liu et al., 2018. Case report China.	1 female. Age: 10 years. Genetically confirmed PTHS diagnosis.	Refractory epilepsy, episodes of cardiac dysrhythmia previously misdiagnosed as myocarditis. Daily episodes of tachypnoea followed by transitory breathing arrest and cyanosis. Re-occurring respiratory tract infection. Some orbital and brain structure abnormalities in MRI.	ASM. Introduction of VPA and later LEV and CZP were added. Follow-up period not reported.	Complete seizure control. No reported side effects. Outcome measure not reported.	100
Matricardi et al., 2022. Case series. Italy.	21 participants: 11 females, 10 males. Age range: 2–35 years (Mdn 11:4). All participants had genetically confirmed PTHS diagnosis.	History of epileptic seizures (100%). Seizure semiology and frequency varied across participants. Onset of seizures: Mdn 2 years, range: 5 months to 8 years. Episodes of breathing abnormalities in (47.6%). Features of ASD (62%) including all participants younger than 5 years, hypotonia (47.6%), stereotypic and repetitive movements (57.2%), developmental coordination disorder (38%), sleep disturbances (19%), behavioural disorders with aggressiveness (14.3%). Postnatal microcephaly (40%) and strabismus (25%). No major congenital malformations were reported.	ASM. 20/21 (95,2%) of participants received ASMs: 10 (47.6%) were on polytherapy. The most prescribed ASMs were VPA − 17/20 (85%), LEV − 8/20 (40%), BZP − 8/20 (40%), and LTG − 5/20 (25%). Follow-up period not reported.	The perceived effectiveness varied. At the end of follow-up, nine patients (42.8%) achieved seizure freedom (seizure control > 12 months). VPA was the drug which most frequently was considered to reduce seizure frequency (5/17). No reported major adverse events or seizure worsening. Outcome measures reported: medical records including video-EEG or caregiver epilepsy diaries.	100
Sulentic et al., 2018. Case report. Croatia.	1 male Age: 24 years. Genetically confirmed PTHS.	Drug-resistant epileptic seizures from the age of 4. Lennox–Gastaut syndrome. MRI post-ischemic porencephalic changes in right occipital cortex. Feet equinovarus deformity and scoliosis.	Neurosurgical treatment, anti-seizure medication (LEV, CZP, oxcarbazepine, VPA) in combination with VNS after several non-satisfactory medical try-outs including ketogenic diet. Follow-up 2 years after neurosurgical treatment.	Improved seizure control. Outcome measure not reported.	56/63 No adverse effects reported. Patient demographic and current clinical condition not sufficiently described.
Yamada et al., 2020. Case report. Japan.	1 female. Age 22 years. PTHS, genetical confirmation not reported.	Refractory epileptic seizures with frequent generalized tonic seizure cluster during sleep and epileptic apnea detected through EEG monitoring and PSG. Breath holding followed by deep expiration during wakefulness. Mild atrophy of brain structures, dysphagia, re-occurring aspirational pneumonia.	ASM. An additional medical treatment with PB and KBr was introduced. Follow-up: approximately one year after intervention started.	Improved seizure control. Epileptic apnea during sleep persisted. Outcome measure reported: overnight video-EEG.	88/100 No adverse effects reported.
Zhao et al.,^2^ 2024. Case series. China.	1 participant. Age: 7 years. Genetically confirmed PTHS diagnosis.	Frequent episodes with complex movements followed by autonomic symptoms. Abnormal EEG.	Ketogenic diet after adverse reactions to ASMs. Follow-up: three months after start of intervention.	Improved seizure control after 3 months. Outcome measures not reported.	83/94. Clinical information, outcomes and demographics not sufficiently described.
**Breathing problems**					
Gaffney & McNally, 2015. Case report. Ireland.	1 male. Age: 15 years. PTHS, genetical confirmation not reported.	Episodes of hyperventilation followed by apnoeas. Microcephaly.	Pharmacological treatment for breathing problems. Treatment with acetazolamide for breathing problems. Follow- up: > 2 years after start of intervention.	Clear decrease in frequency and severity of episodes of hyperventilation and apnea. No side effects. Outcome measure reported: parental reports.	50/63 Demographics, clinical conditions, diagnostic methods, post clinical conditions and adverse effects not reported.
Maini et al., 2012. Case report Italy.	1 female. Age: 7 years. Genetically confirmed PTHS diagnosis.	Non-epileptic episodes of hyperventilation alternating with apnoeic episodes, sometimes associated with cyanosis during wakefulness.	Pharmacological treatment for breathing problems. Introduction of VPA after non-effective treatment with DZP. Follow-up at 7 months after the start of intervention.	Clinical improvements. Improvement of apnoeas and of saturation. Hyperpneic episodes persisted. Outcome measure reported: polysomnography.	100
Verhulst et al., 2021. Case report. Belgium.	2 males. Ages 21 (A) and 9 (B) years. Both had genetically confirmed PTHS diagnosis.	Participant A: Daily episodes of hyperventilation, apnea, and cyanosis exacerbated by stress. Epilepsy treated with VPA. No signs of brain stem abnormalities. Participant B: Daily episodes of serious hyperventilation followed by apnea and syncope. No signs of brain stem abnormalities.	Pharmacological treatment for breathing problems with acetazolamide. Follow-up: 4 weeks/5 months.	Particpiant A: Improvement of apnoeas and of saturation. Participant B: Improvement of apnoeas and of oxygen saturation. A break in treatment resulted in new episodes which disappeared when treatment was restarted. Outcome measure reported: polygraphic monitoring.	88/100 No adverse effects reported.
**Psychiatric issues**					
Istanbullu et al., 2023. Case report. Turkey.	1 female Age: 7 years Genetically confirmed PTHS diagnosis.	Hyperactivity, sleep disturbances, anxiety symptoms, restlessness. ASD, microcephaly and corpus callosum hypoplasia.	Psychopharmacological treatment with aripiprazole and escitalopram for anxiety symptoms and quetiapine to improve sleep after several non-satisfactory try-outs. Follow-up: not reported.	Improvements in sleep, diminished hyperactivity, decreased frequency of abnormal breathing spells and enhancement of social communication. Slight improvement of adaptive behaviour. Outcome measures reported: parental reports, VABS to measure adaptive behaviour.	100
Lambrechts et al., 2018. Case report. Belgium.	2 females (sisters). Ages 14 (A) and 12 (B) years. PTHS, Genetical confirmation not reported.	Participant A: Agitation, anxieties associated with changes in routine, anhedonia. Features of ASD with poor contact, verbal perseveration, and echolalia. Hypotonia. Participant B: Frequent temper tantrums, sleeping problems, hyperactivity, aggressive behaviour, self-injury, excessive laughing. ASD, short attention span, stereotypies.	Psychopharmacological treatment. Participant A: Medication for psychiatric symptoms with aripiprazole at low dose after several non-satisfactory medical try-outs. Follow-up: one year. Participant B: Medication for psychiatric symptoms. Introduction of risperidone and aripiprazole, followed by several adjustments. Follow-up: approximately 2 years after start of intervention.	Participant A: Reduction of anxiety and anhedonia persisting at follow up. Fatigue and excessive sleeping were reported as side effects. Participant B: Decrease in agitation, shouting, aggression and sleep problems while motor stereotypies remained. Several dose adjustments. Outcome measures not reported.	100
Vieira et al., 2021. Case report: Brazil.	1 female. Age: 23 years, PTHS, genetical confirmation not reported.	Catatonia, problematic behaviors following changes in routine. Epilepsy, repetitive lower urinary tract infections, non-verbal ASD.	ECT treatment. Treatment ECT against catatonia after non-satisfactory treatment with antidepressants. Follow-up: 15 days after end of intervention.	Patients’ behavior returned to pre-catatonia patterns according to Bush-Francis Catatonia Rating Scale. No serious side effects were reported although potential post-ECT hyperthermia was considered.	94/100 Diagnostic methods not clearly described.
**Other**					
Casey et al., 2018. Case report. Canada.	1 male. Age: 4 years. Genetically confirmed PTHS diagnosis.	Ataxia and low muscle tone, fatigue-like symptoms and sedentary behavior. Sparse social interaction, repetitive behaviors, agitation/anxiety, gastro-esophageal reflux, reoccurring flu and pneumonia.	An adapted walking intervention to increase mobility. Five weekly one-hour sessions for 12 weeks. Follow-up: three months after end of intervention.	Improvement on several social, physical and motor measures. At follow up some effects remained whilst other effects decreased or diminished. Outcome measures reported: GAS to evaluate social interaction, physical activity, and physical health; MAP and mobility goals to assess mobility.	100
Malik et al., 2023. Case report. USA.	1 female. Age: 18 years. Genetically confirmed PTHS diagnosis	Common carriable immunodeficiency, frequent hospitalizations for infection, feeding issues, hypoventilation, sleep apnea requiring BiPAP. Seizures, microcephaly.	Immunoglobulin replacement therapy to prevent infections. Follow-up: > 3 years after start of intervention.	Decreased hospital admissions and number of pneumonias compared to before treatment. Outcome measures not reported.	94/100 Patient demographics not sufficiently described.
Reaney & Collins, 2024 Case report. Ireland.	1 male. PTHS diagnosis	Visceral hyperalgesia resulting in several hospitalisations for treatment including intravenous clonidine, chronic constipation, previous rectal prolapse, severe gastrointestinal issues and nutritional deficits despite surgical intervention of the colon and gastrostomy. Epilepsy, chronic aspiration secundary to excess orapharyngeal secretations, nonobstructive hydrocephalus.	Individualised pain management plan and clonidine patches for visceral hyperalgesia. Follow-up: >7 months after start of intervention.	Pain could be managed at home without intravenous clonidine. Increased tolerance for food given enterally and orally. Outcome measures reported: behavioural observation and parental reports.	94 Participant demograhics not sufficiently described.
Zhao et al.,^3^ 2024. Case series. China.	8 participants: 1 female, 7 males. Age range: 1 year – 5 years, (Mdn 1:7) All participants had genetically confirmed PTHS diagnosis.	Constipation, (*n* = 8/8), low food tolerance (*n* = 4/8), indigestion (2/8), hyperactivity/ASD (2/8), hyperactivity or lower concentration (3/8).	Carbohydrates diet (SCD) and anti-candida supplements targeting digestive health. Follow-up: maximum 3 months.	Significant improvements in digestive function, reduced constipation, and improved mental clarity. Enhancements in speech, social communication ability and emotional expression. Outcome measures not reported.	83/94. Clinical information, outcomes and demographics not sufficiently described.

^1^Symptoms included in the diagnostic criteria for PTHS suggested by Zollino et al. (2018) are not reported when not targeted in the intervention

^2^When there was disagreement between the two raters, both raters’ assessments are presented. JBI critical appraisal checklists for case reports respectively case series were used to conduct the quality appraisal

^3,4^ In a case series by Zhao et al. which included 47 patients diagnosed with PTHS, an intervention was related to outcomes regarding seizures^3^, and gastric problems^4^. These are presented under separate headings in this table but refer to one study in the running text

Abbreviations: ASD = autism spectrum disorder, ASM = Antiseizure medications, BZP = Benzodiazepine, CBZ = Carbamazepine, CLB = Clobazam, CZP = Clonazepam, DZP = Diazepam, ECT = Electroconvulsive therapy, EEG = Electroencephalogram, ESM = Ethosuximide, GAS = Goal Attainment Scaling; Jones et al., 2006, GDD = global developmental delay, ID = intellectual disability, KBr = Potassium Bromide, LEV = Levetiracetam, LTG = Lamotrigine, MAP = Mobility, Ability, Participation, MRI = Magnetic resonance imaging, PB = Phenobarbital, PMP = Perampanel, PSG = Polysomnography, PTHS = Pitt-Hopkins syndrome, RUF = Rufinamide, TMP = Topiramate, VPA = Valproic acid, ZNS = Zonisamide

### Participant characteristics

In total, 84 participants with PTHS were included in the review. A genetic confirmation of the PTHS diagnosis was reported for 79 of the 84 participants. A majority of the studies had one or two participants (14/16). The age span of the participants ranged from 2 to 35 years and there were both males and females included in the studies. Regarding other reported comorbid conditions, a majority of the participants had epilepsy including refractory epilepsy. Several participants had language delays or absence of speech, and several were reported as having severe or profound intellectual disabilities. Other reported comorbid conditions were ASD, psychomotor delays, hyperventilation followed by apnea, visual and hearing impairments, fatigue-like symptoms, sleeping problems and problematic behaviours.

### Study designs and methods used in the studies

A case report design was used in all but two studies, which were both case series, including 21 and 47 participants, respectively. Only 10 of the included studies reported outcome measures or described the methods used to assess intervention effects. The outcomes were mainly reported as increase, decrease or full control of symptoms. Of these studies, two reported that the data were derived from parental reports, while one relied on medical records without providing further methodological details. One study reported that they used medical records and behaviour observation. Three of the studies used EEG or video-EEG to evaluate the effect of seizure treatment.

Polygraphic monitoring was used in two studies to evaluate the effect of breathing-related interventions. In one study [16], goal attainment scaling (GAS), mobility ability participation assessment and predefined mobility goals were employed to assess improvements in social participation, mobility and physical health. Vieira et al. [17] used the Bush-Francis Catatonia Rating Scale to evaluate the effect of Electroconvulsive therapy. Furthermore, VABS was reported as a tool to follow up adaptive behaviour (18).

### Types of interventions and reported benefits or effectiveness

Pharmacological interventions were reported in 13 of the 16 studies. Six studies investigated treatments for epilepsy and seizures, three examined medications targeting breathing abnormalities, two evaluated treatments of psychiatric symptoms, one assessed immunoglobulin replacement therapy for the prevention of pneumonia, and one evaluated an individualised pain management plan for a patient with visceral hyperalgesia. Of the remaining three studies, one used electroconvulsive therapy (ECT) for the treatment of catatonia, one investigated the ketogenic diet for seizure management in combination with dietary interventions aimed at improving gastrointestinal health and one evaluated a walking-based intervention.

Six case reports describe positive results of ASM. In one study (19), the addition of perampanel (PMP) to valproate (VPA) was associated with improved seizure control following several prior medication trials that had failed to achieve the desired effect. In a case report by Liu et al. [18] a young female was reported to be seizure-free following the addition of levetiracetam (LEV) and clonazepam (CZP) to VPA. Yamada et al. [4] describe a young woman with an increasing frequency of seizures. Electroencephalographic (EEG) monitoring and polysomnography revealed frequent clusters of generalized tonic seizures during sleep, associated with epileptic apnea. Treatment with phenobarbital (PB) and potassium bromide (KBr) reduced the frequency of clusters of generalized tonic seizures although epileptic apnea during sleep persisted. Kirikae et al. [19] report a dramatic decrease and diminishing epileptic seizures of a toddler with West syndrome after Topiramat (TMP) was administered. Several previous attempts had been made without positive results. At 1 year and 8 months, psychomotor and behavioural improvements were observed, and EEG showed resolution of hypsarrhythmia. In a retrospective group study 20/21 participants received ASM. Monotherapy was used in half of the cases while the other half received polytherapy. VPA was the most commonly prescribed ASM followed by LEV, benzodiazepine (BZP), and lamotrigine (LTG) with mixed results. The medicines that were found effective were VPA (5/17, 29.4%), LEV (1/8, 12.5%), carbamazepine (CBZ) (2/4, 50%), TMP (2/4, 50%), ethosuximide (ESM) (1/1, 100%), clobazam (CLB) (1/3, 33.3%), rufinamide (RUF) (1/1, 100%), and zonisamide (ZNS) (1/1, 100%). Only one study reports that ASM was discontinued due to side effects [20]. Calle-Lopez [21] report cosmetic side effects in the form of curly hair from PMP. Zhao et al. [20] report a reduction in seizure frequency of over 90% in one patient aged 7 years and 1 month after three months of ketogenic diet treatment.

Three case reports document the treatment of four participants with breathing-related conditions. Maini et al. [22] report a reduction of apneic or hypopneic episodes and improvement of saturation values after VPA was added to an ineffective treatment of diazepam (DZP) which also induced excessive sleepiness. Sub-continuous abnormal breathing patterns during wakefulness persisted but apneic or hypopneic episodes were decreased and saturation values improved. In two case reports [23, 24] treatment with acetazolamide was reported to reduce the severity and frequency of episodes of hyperventilation and apnea and improve saturation values in a total of three participants. For two of the three participants, the positive effect was immediate.

Two studies describe treatment with aripiprazole for psychiatric and behavioural issues. In one case, treatment with aripiprazole, escitalopram and quetiapine was reported to improve anxiety symptoms and sleep problems in a 7-year old girl [25]. These symptoms were also associated with hyperventilation episodes. Various medication combinations and dose adjustments were explored but proved ineffective or produced adverse effects. Lambrechts et al., [26] describe psychopharmacological treatment in a case report with two young sisters. In one of the females, a low dose reduced anxiety and anhedonia. After dose regulations to decrease side effects (fatigue and falling asleep early), the positive effects remained at a one year follow up although she still fell asleep early. For the other female, a combination of risperidone and aripiprazole resulted in a decrease in sleep problems, agitation, self-injury and other problematic behaviour while motor stereotypies remained. Several adjustments of the dose were described. Non-medical adjustments were not reported.

One case report describes the use of electroconvulsive therapy (ECT) for the treatment of catatonia in a young female patient [17], with behavioral changes developing after sudden routine disruptions related to the COVID-19 pandemic. After approximately one year, she was admitted to a psychiatric ward with symptoms including agitated behavior characterized by unresponsiveness, posturing, stereotypic movements, insomnia, waxy flexibility, and minimal food intake accompanied by significant weight loss. Treatment with antidepressant medication showed little to no effect; however, following 13 sessions of electroconvulsive therapy (ECT), the patient’s behavior returned to pre-catatonic patterns and remained stable at 22-day follow-up. No serious adverse effects were reported, although possible post-ECT hyperthermia was considered.

In Malik et al. [27] a young female with common carriable immunodeficiency in addition to PTHS received immunoglobulin replacement therapy to prevent pneumonias. The treatment was described to reduce hospital admissions from 10 per year to 5–6 per year. After two breaks in treatment due to high burden of therapy she had serious pneumonias and the therapy was resumed.

Reaney et al. [28] describe a case with an early adolescent boy with a history of severe constipation. He had surgical intervention due to colon dilations and obstruction. Despite these interventions, severe gastrointestinal problems persisted. He also had visceral hyperalgesia with pain that was not relieved by simple or opioid analgesia and required several hospital admissions. Through an individual pain management plan and thorough follow-ups the pain was generally well managed and could be managed at home.

Casey et al. [16] report an adapted walking intervention, consisting of five weekly one-hour sessions for 12 weeks with a 4-year old boy. During the sessions, the Upsee mobility device [29] was used and an assessment and therapy program, Movement Ability Participation, (MAP) related to the Upsee guided the intervention. At the termination of the 12-week intervention there were significant effects on several social, physical and motor measures. Goals set according to Goal attainment scaling (GAS) [30] concerning social interaction, physical activity and physical health were surpassed at 12 weeks. He had also reached mobility goals and increased ability in head control, weight bearing and walking patterns. At follow-up, the walking outcome is reported inconsistently. Specifically, in a table the coded stage label (maintains stepping pattern with full help) does not align with the accompanying descriptive criterion (maintains stepping pattern with some help). During the intervention the participant´s ability to participate in play and social interaction increased. However, at 24-week follow-up, several initial gains were not maintained, except for hand function.

### Quality appraisal

Study quality was assessed using the Joanna Briggs Institute (JBI) checklists for case reports and case series [13, 14]. The raters assigned an average of 91% of the maximum possible score across all studies. Adverse effects were not reported in five studies. Given that most studies were case reports, some variation in reporting detail was expected. Nonetheless, all studies were considered sufficiently transparent to inform clinical insights (see Table 3).

## Discussion

This scoping review aimed to examine the existing literature on interventions targeting challenges experienced by individuals with PTHS and to identify gaps in the current knowledge base. PTHS is diagnosed based on clinical symptoms, particularly the presence of unique facial features, severe intellectual disability, communication challenges, epilepsy, and breathing regulation abnormalities. Consequently, the syndrome has a profound impact on physical health as well as on everyday functioning. Individuals with PTHS are also considered to be at heightened risk of developing mental health problems [6, 8].

A total of sixteen studies met the inclusion criteria, published between the years 2012 and 2024. The findings primarily reflect the medical needs of this population, as most of the interventions focused on epilepsy or breathing difficulties, and the majority were pharmacological in nature. Most studies employed a case report design with only one or two participants. Systematic methods to evaluate the effects were rarely reported. For instance, epilepsy diaries [31] and sleep diaries [32] are commonly used clinical tools to systematise the data collection; however, only one study in the current review utilised an epilepsy diary. These methodological gaps highlight the need for more rigorous, controlled research to minimize bias and enhance the generalizability of findings.

It should nevertheless be emphasised that intervention research targeting challenges in individuals with PTHS is still at an early stage. Given the small number of studies available, all included publications may be regarded as important contributions to the field. By mapping the current evidence, this review seeks to stimulate further research and thereby support the development of more effective interventions targeting challenges in individuals with PTHS. Overall, the included studies highlight the importance of thorough follow-ups and individualized care plans, as evidenced by the frequent adjustments to doses and treatment regimens.

The predominance of case reports and small case series illustrates the challenges of establishing an evidence base sufficient for conventional evidence-based clinical guidelines in PTHS. Consequently, clinical management relies on the best available evidence together with expert consensus. Across the different clinical domains reviewed, our findings are broadly consistent with the international consensus recommendations by Zollino et al. [1], particularly regarding the importance of individualized management, multidisciplinary follow-up, and the management of epilepsy, respiratory dysfunction, gastrointestinal complications, and motor functioning. In line with this, clinical practice guidelines are currently being developed using a consensus methodology by the ERN ITHAKA Clinical Guidelines Workgroup. At the same time, our review highlights several areas that remain underrepresented in the intervention literature, including communication, learning, behaviour, participation in everyday activities, and family outcomes. Developing PTHS-relevant knowledge into these domains will likely draw on insights from other (rare) neurodevelopmental conditions.

The current review identified seven case reports and two case series on epilepsy in individuals with PTHS, highlighting current clinical experiences and treatment strategies. The findings reaffirm the clinical heterogeneity of PTHS epilepsy [6], with epileptic phenotypes ranging from mild focal epilepsy [5] to severe encephalopathies like West syndrome and Lennox-Gastaut syndrome [5, 19, 33]. This variability in seizure characteristics and progression emphasizes the challenges in managing epilepsy in PTHS patients.

Also, the findings of the review indicate that treatment strategies for epilepsy in patients with PTHS largely align with those used in the broader epilepsy population. Approximately 60% of patients received polytherapy, and VPA was the most frequently prescribed medication, either as monotherapy or in combination with LEV, LTG, or CZP. In treatment-resistant cases, newer-generation antiseizure medications (ASMs) such as PMP and TMP were reported to be effective, consistent with previous findings [34–36]. However, the risk of interactions between drugs and cumulative side effects remains a key concern in polytherapy regimens [34, 35].

Non-pharmacological treatment options were described in only four of the included studies, namely a walking-based mobility intervention, vagus nerve stimulation (VNS), corpus callosotomy, and ketogenic diet [5, 16, 20, 33]. Although the reported outcomes were heterogeneous, these findings suggest that alternative therapeutic modalities may be valuable options for drug-resistant epilepsy, aligning with recent evidence from broader populations [37–40]. Nonetheless, systematic studies assessing the efficacy and safety of these interventions in PTHS remain scarce.

Despite non-epileptic breathing abnormalities being reported as frequently, or more so, than epilepsy in PTHS [6], only three case studies have explored interventions for this symptom. One possible reason is that these breathing disturbances are typically brief (2–5 min) and considered benign [1]. However, they may lead to central hypoxia, cyanosis, and, in some cases, chronic hypoxia [2], which could potentially harm lung function. Aerophagia, associated with these abnormalities, may in rare cases contribute to serious gastrointestinal complications [41]. In one case, VPA was found to reduce apnea/hypopnea, a notable finding given its common use in seizure management but limited documentation for respiratory dysfunction in PTHS. A prospective pilot study by Hamed et al. [42] demonstrated that VPA improved hyperventilation and cyanosis in children with similar respiratory patterns, suggesting a potential therapeutic mechanism relevant to PTHS. The underlying pathophysiology of hyperventilation and apnea in PTHS remains unclear, though autonomic dysfunction has been proposed as a contributing factor [2, 3, 43–45]. The neuroprotective properties of VPA [46, 47] may provide a possible mechanistic explanation.

Two case reports [23, 24] described immediate reduction of hyperventilation and apnea following initiation of acetazolamide treatment. This approach is informed by practices in high-altitude medicine and acute mountain sickness [48], as well as the treatment of non-hypercapnic central sleep apnea [49, 50], highlighting its potential translational relevance for PTHS-related respiratory dysfunction.

In another case report by Istanbullu et al. [25], psychopharmacological treatment for anxiety related to hyperventilation was found to reduce the frequency of abnormal breathing episodes. This aligns with previous studies, which have reported that hyperventilation episodes are often linked to emotional distress, anxiety, or agitation [2, 6, 51].

Gastrointestinal manifestations are highly prevalent among individuals with PTHS, with constipation being the most frequently reported symptom, showing an age-related increase in prevalence (reported in up to 100% of cases) [6]. Autonomic dysfunction has been proposed as the underlying cause of impaired intestinal motility [52]. Chronic constipation may, in some individuals, be further complicated by aerophagia and abdominal bloating [1, 53]. Isolated cases of PTHS co-occurring with Hirschsprung disease and intestinal malrotation have also been described [6, 54]. These conditions, together with aerophagia, have been associated with severe gastrointestinal complications [1, 41].

Pain assessment and management present additional challenges in individuals with PTHS, as pain may be secondary to a range of gastrointestinal pathologies [53]. Visceral hyperalgesia has also been documented in isolated cases [28], where the introduction of an individualized pain management plan involving transdermal clonidine proved effective in achieving symptom relief and reducing hospital admissions.

In recent years, attention has increasingly turned toward the role of the gut microbiome in gastrointestinal health and disease. The concept that the gut microbiome plays a pivotal role in metabolism, immune regulation, and neurobehavioral function has become well established. The gut–brain axis refers to the bidirectional communication between the central nervous system (CNS) and the gastrointestinal tract [55]. Biomedical interventions targeting the gut microbiome, including dietary modulation, have been proposed as potential therapeutic strategies. One of the identified studies [20] highlighted microbiome-targeted dietary interventions as promising adjunctive or alternative approach in PTHS, as it has been suggested for other neurodevelopmental and psychiatric disorders including autism spectrum disorder (ASD), depression, Rett syndrome, and Angelman syndrome [56]. Specifically, comparative studies have demonstrated distinct differences in the gut microbiota composition between individuals with such neurodevelopmental conditions and healthy controls. Interventional trials that modify the microbiome through the administration of probiotics, prebiotics, or dietary changes have shown improvements in both psychopathological and gastrointestinal symptoms, including constipation [57].

Various immunodeficiency-related abnormalities have been described in association with deletions involving the long arm of chromosome 18 (18q), proximal to the TCF4 gene [58]. Although recurrent respiratory infections—such as otitis media, tonsillitis, and bronchitis—and urinary tract infections have been reported in approximately one-third of individuals with PTHS, particularly during childhood, clinically significant immunological abnormalities are rarely identified [6]. However, it should be noted that immunological testing is seldom performed in this population [1]. One single case report in our review described repeated immunoglobulin substitution therapy for common variable immunodeficiency in a patient with PTHS [27], suggesting that immune dysfunction, although uncommon, may occur in specific cases and warrants further investigation.

Three of the studies examined interventions targeting psychiatric, neuropsychiatric, or behavioral difficulties such as unresponsiveness, anxiety, hyperactivity, and sleep disturbances. Pharmacological treatments and electroconvulsive therapy (ECT) were reported to yield improvements in these areas. However, research on behavioral and psychosocial interventions for individuals with PTHS remains very limited. There is a need for studies evaluating their potential effectiveness, either as stand-alone strategies or in combination with medical treatments. More broadly, research is also warranted on approaches that promote general well-being and quality of life, thereby potentially enhancing resilience to psychiatric and behavioral challenges.

The intervention in Casey et al. [16], utilizing a walking device (Upsee), demonstrated improvements in mobility, physical activity, and social interaction. Previous research underscores the importance of interventions aiming at enhancing gross motor skills, which not only support motor development but also mitigate the risk of orthopedic complications [6]. Physical activity has been shown to benefit individuals with developmental disabilities by enhancing health, aerobic capacity, gross motor skills, and overall satisfaction among both participants and their parents [59]. In Casey et al. [16], the use of an upright position also facilitated social interaction in daily contexts, thereby fostering increased participation in everyday activities. However, the results indicated that following the cessation of the intervention (and the reduction in the use of the Upsee device), there was a notable decline in social interaction and play participation. This suggests that physical aids may provide individuals with the necessary conditions to engage in physical activity for extended periods, thereby enhancing their ability to interact and participate in daily life. To ensure sustainability, resource efficiency, and applicability across diverse settings, including home and educational environments, it is essential to integrate such interventions into daily routines.

### Methodological considerations beyond case studies

This scoping review highlights the methodological limitations of current research on interventions for PTHS. While case studies offer valuable clinical insights, they cannot establish causal relationships. To enable valid causal inferences, we believe research must employ designs that systematically manipulate independent variables and repeatedly measure dependent variables [60]. Thus, the reliance on descriptive studies limits the possibility of developing evidence-based clinical recommendations based solely on empirical data. Nevertheless, for rare disorders such as PTHS, consensus-based recommendations remain an essential framework for clinical management until stronger evidence becomes available. A viable alternative, in some cases and in some situations, is the use of single-case experimental designs in clinical research and care. Such designs may facilitate experimental control and support more robust empirical conclusions that can contribute to a cumulative evidence base [61]. These methods are especially apt in the context of rare conditions as they are designed to evaluate interventions for a single individual or a small number of participants [60]. Furthermore, single-case experimental designs are frequently employed in research regarding building and extending repertoires in areas such as communication, participation in activities and other adaptive behaviors [62].

### Limitations of the current review

This review draws on four major databases and manual reference screening, which may have resulted in the omission of studies not indexed in these sources (including grey literature). In addition, the exclusion of Pitt–Hopkins–like syndromes, although intended to maintain diagnostic specificity, may have further narrowed the number of relevant studies. Despite these caveats, the current scoping review does make a contribution by charting existing peer reviewed literature on interventions targeting challenges experienced by individuals with PTHS as well as identifying gaps in the current knowledge base.

## Conclusion

Given the wide range of physical health issues associated with Pitt Hopkins syndrome, studies aimed at improving management in areas such as physical activity, gastrointestinal problems [41], and dental care [63] are crucial. However, we would like to emphasise the importance of intervention research in other domains, including communication, learning, behavior, and participation in daily activities, as these could significantly improve well-being and quality of life. Additionally, exploring the impact of the diagnosis on families of individuals with PTHS would be a valuable area for future research and investigation.

## Data Availability

Data and materials and available upon request.
